# A Newly Created Meso-, Micro-, and Nano-Scale Rough Titanium Surface Promotes Bone-Implant Integration

**DOI:** 10.3390/ijms21030783

**Published:** 2020-01-25

**Authors:** Masakazu Hasegawa, Juri Saruta, Makoto Hirota, Takashi Taniyama, Yoshihiko Sugita, Katsutoshi Kubo, Manabu Ishijima, Takayuki Ikeda, Hatsuhiko Maeda, Takahiro Ogawa

**Affiliations:** 1 Weintraub Center for Reconstructive Biotechnology, Division of Advanced Prosthodontics, UCLA School of Dentistry, Los Angeles, CA 90095-1668, USAmhirota@yokohama-cu.ac.jp (M.H.); manab612@gmail.com (M.I.); ikeda.takayuki@nihon-u.ac.jp (T.I.);; 2 Department of Oral Pathology, School of Dentistry, Aichi Gakuin University, 1-100 Kusumoto-cho, Chikusa-ku, Nagoya, Aichi 464-8650, Japan; 3 Department of Oral Science, Graduate School of Dentistry, Kanagawa Dental University, 82 Inaoka, Yokosuka, Kanagawa 238-8580, Japan; 4 Department of Oral and Maxillofacial Surgery/Orthodontics, Yokohama City University Medical Center, 4-57 Urafune-cho, Yokohama, Kanagawa 232-0024, Japan; 5 Department of Orthopedic Surgery, Yokohama City Minato Red Cross Hospital, 3-12-1 Shinyamashita, Yokohama, Kanagawa 231-8682, Japan

**Keywords:** acid-etching, bone-implant integration, dental implants, hierarchical morphology, meso–micro–nano roughness, osseointegration, orthopedic implants, titanium surface

## Abstract

Titanium implants are the standard therapeutic option when restoring missing teeth and reconstructing fractured and/or diseased bone. However, in the 30 years since the advent of micro-rough surfaces, titanium’s ability to integrate with bone has not improved significantly. We developed a method to create a unique titanium surface with distinct roughness features at meso-, micro-, and nano-scales. We sought to determine the biological ability of the surface and optimize it for better osseointegration. Commercially pure titanium was acid-etched with sulfuric acid at different temperatures (120, 130, 140, and 150 °C). Although only the typical micro-scale compartmental structure was formed during acid-etching at 120 and 130 °C, meso-scale spikes (20–50 μm wide) and nano-scale polymorphic structures as well as micro-scale compartmental structures formed exclusively at 140 and 150 °C. The average surface roughness (Ra) of the three-scale rough surface was 6–12 times greater than that with micro-roughness only, and did not compromise the initial attachment and spreading of osteoblasts despite its considerably increased surface roughness. The new surface promoted osteoblast differentiation and in vivo osseointegration significantly; regression analysis between osteoconductivity and surface variables revealed these effects were highly correlated with the size and density of meso-scale spikes. The overall strength of osseointegration was the greatest when the acid-etching was performed at 140 °C. Thus, we demonstrated that our meso-, micro-, and nano-scale rough titanium surface generates substantially increased osteoconductive and osseointegrative ability over the well-established micro-rough titanium surface. This novel surface is expected to be utilized in dental and various types of orthopedic surgical implants, as well as titanium-based bone engineering scaffolds.

## 1. Introduction

Endosseous implants have become an important treatment modality in medicine and dentistry [1,2,3]. In particular, orthopedic and dental implants such as plates, screws, and joint reconstructive prostheses are commonly used in the treatment of bone fractures, osteoarthritis, bone defects after tumor resection, and other bone and joint disorders and pathogeneses [4,5,6,7,8]. In the field of dental implants, titanium and its alloys have been used as implant materials for 50 years, ever since the concept of osseointegration was established by Brånemark et al. [9]. In order to achieve better osseointegration, various implant materials have been developed. Surface characteristics of implants—one of the principal factors affecting the process of osseointegration [10]—have been a topic of growing interest for decades. Various titanium surfaces with different micro-scale topography have been developed and studied [11,12,13,14]; however, many challenges remain, including difficulties in determining whether one particular implant surface is better than the others [15].

The micron-scale topography of a titanium surface is commonly created by acid-etching, sandblasting, or a combination of both [16]. In addition, the micro-roughness of titanium alloys has also been reported [17]. Numerous studies have shown that such micro-scale topography promotes the adhesion, differentiation, and extracellular matrix formation and mineralization of osteoblasts [18,19,20,21,22]. In contrast, several studies have shown that such osteoblast attachment, spreading, and proliferation can be negatively affected by micro-scale topography [19,20,23]. Although it is clear that the micro roughness of the implant surface promotes the overall process of osseointegration, especially in the early phases of healing [24], it remains necessary to further promote differentiation and—more importantly—the potential alleviation of the inhibitory effects on osteoblast attachment, spreading, and proliferation.

Nano-scale design is expected to offer certain benefits for implant materials. It has many advantageous characteristics for cell attachment and spreading [25,26], aiding in the production of more extracellular matrix proteins and enhancing the mechanical interlocking of biomaterials [27]. Moreover, cell-to-cell and cell-to-biological tissue interactions including surface sensing and recognition occur at the molecular level in nano-scale models, as seen in cells interplaying with the nanofeatures of peptide folding, protein complexing, collagen binding, enzymes, and antibodies [28,29]. However, these studies have been conducted using materials other than titanium. It is unclear whether and to what extent the nano-surface improves the osteoconductive and osseointegrative ability of biomaterials [30]; more importantly, nano-scale features have yet to be successfully formed on titanium materials or commercial implant products [31,32]. Although reports exist on the creation of nano-scale titanium surfaces [14,33,34], the nano-features did not present distinct or defined appearance. In addition, it is technically difficult to control nano-scale structure, prompting the need for further studies.

To the best of our knowledge, the present study is the first to create meso-scale structures on titanium surfaces. In addition to the well-known micro-scale structure, we successfully created a completely new titanium surface having meso- and nano-scale structure. We hypothesized that this new meso-, micro-, and nano-scale roughness enhances osseointegration by increasing bone–titanium interlocking and further promoting osteoblast differentiation beyond the capacity of surfaces with micro-scale roughness alone. Therefore, the purpose of this study was to investigate the biological and osseointegration capabilities of our newly created meso-, micro-, and nano-scale rough titanium surface and to control and optimize the meso-scale structure for better biological performance.

## 2. Results

### 2.1. Morphology of Titanium Surfaces

**Figure 1:**
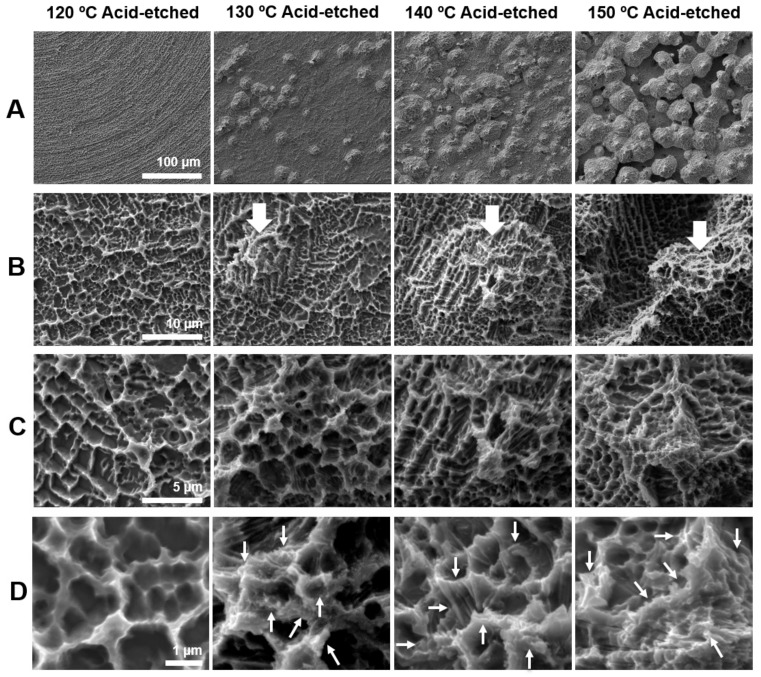
Surface morphology of the titanium disks used in this study. Scanning electron microscopy (SEM) photographs showing surface roughness after acid-etching (H_2_SO_4_) at 120, 130, 140, and 150 °C. (**A**) 500× magnification, (**B**) 5000× magnification, (**C**) 10,000× magnification, and (**D**) 30,000× magnification. Scale bar = (A) 100 μm, (B) 10 μm, (C) 5 μm, and (D) 1 μm.

Figure 1 shows the morphology of acid-etched titanium surfaces at different temperatures of 120, 130, 140, and 150 °C evaluated by scanning electron microscopy (SEM). The greatest contrast was seen in the lowest magnification images (Figure 1A). Titanium surfaces acid-etched at 120 °C showed no recognizable structures at lowest magnification, whereas those etched at other temperatures showed spike-like projecting structures across the surface. The sizes of the spikes were in the meso-scale and ranged from 10 to 80 μm according to the SEM images. The density and size of the spikes were greater on titanium surfaces etched at higher temperatures. Higher magnification images clearly showed the spikes more vividly on all surfaces (white arrowheads in B) except for the one etched at 120 °C; moreover, the surface of the spikes began to show microroughness as in other areas of the titanium (Figure 1B). Even higher magnification images configured the microroughness as a compartmental structure consisting of peaks and valleys (Figure 1C). This microrough feature was in common for all surfaces, including the one etched at 120 °C. The highest magnification images revealed nano-scale structures exclusively formed on titanium surfaces etched at 130 °C or higher (Figure 1D). Nano-scale structures were not observed on 120 °C acid-etched titanium, even at the highest magnification. Nanofeature was polymorphic, including but not limited to nano-ridge, nano-nodule, nano-pillar, and nano-compartmental structures (white arrowheads in Figure 1D). Thus, we discovered new titanium surfaces with distinct morphology simultaneously at three different levels of meso-, micro-, and nano-scales.

### 2.2. Roughness Characteristics of Titanium Surfaces

**Figure 2:**
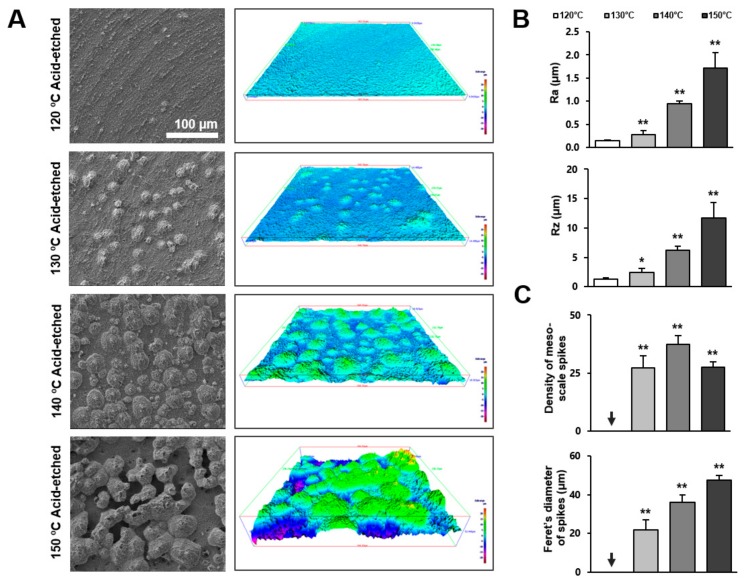
Three-dimensional profile and quantitative topographical evaluations of titanium surfaces. (**A**) Three-dimensional scanning electron microscopy (3D-SEM) images were built by SEM images. (**B**) Profile analysis: Ra (average roughness of profile) and Rz (the peak-to-valley height of the roughness profile within a sampling length) were evaluated. (**C**) The number of meso-scale spikes and Feret’s diameter of spikes were measured. Each value represents the mean ± standard deviation of three sites on the four different surfaces (*n* = 5). * *p* < 0.05, ** *p* < 0.01, with a statistically significant difference when compared to the 120 °C acid-etched surface.

Following qualitative observation by SEM, we further characterized the three-dimensional (3-D) morphology of the titanium surfaces by roughness profiling. Figure 2A is a side-by-side presentation of the SEM images of the four different titanium surfaces and the 3-D color-contrasted images created from the corresponding SEM images. Confirming the SEM observations, titanium surfaces acid-etched at 130 °C or higher showed meso-scale spike-like projections, with their density and size generally increasing with temperature during acid-etching. Interestingly, the density of the meso-spikes appeared to plateau at 140 °C, whereas their size continued to increase. Due to the maximally increased size of the spikes, as well as their substantial density, the inter-spike spaces were less on the 150 °C-acid-etched surface than on the 140 °C-acid-etched surface.

Figure 2B,C shows the quantification of roughness values. The average roughness (Ra) increased in correlation with the acid-etching temperature. The increase was more significant when the temperature rose from 130 to 140 °C. The Ra of the 140 °C- and 150 °C-acid-etched surfaces was 6 and 12 times greater, respectively, than the 120 °C-acid-etched surface. The peak-to-valley roughness (Rz) also increased in correlation with temperature. Supporting the observation mentioned earlier, the density of the meso-scale spikes increased with the acid-etching temperature and plateaued at 140 °C, whereas their Feret’s diameter continued to increase until the temperature reached 150 °C. The sizes of the meso-spikes ranged from 20 to 50 μm according to the Feret’s diameters of the spikes, confirming the generation of meso-scale structures.

### 2.3. Cell Attachment and Spreading Behavior of Osteoblasts

**Figure 3:**
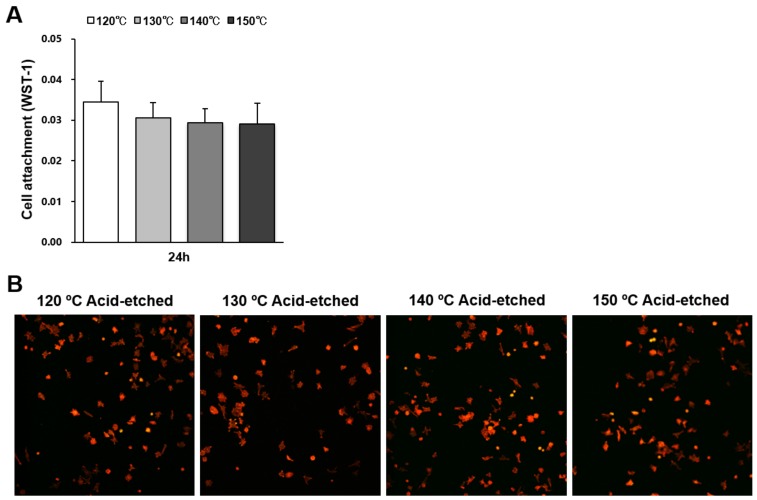
Attachment of osteoblasts on titanium surfaces acid-etched at different temperatures. (**A**) Number of cells attached to each titanium surface during a 24 h incubation, evaluated by a WST-1 assay. No statistically significant differences among the different surfaces were detected. Each value represents the mean ± standard deviation. (**B**) Initial spread of osteoblasts 24 h after seeding on titanium surfaces. Representative confocal microscopy images of cells stained with rhodamine phalloidin for actin filaments (red) and anti-vinculin for vinculin (green).

The number of osteoblasts attached to titanium surfaces during the initial stage of culture was evaluated by water soluble tetrazolium salts-1 (WST-1) assay 24 h after seeding (Figure 3A). There were no significant differences between the different temperatures. Low-magnification confocal microscopy images showing a similar quantity of cells confirmed the WST-1 results (Figure 3B).

**Figure 4:**
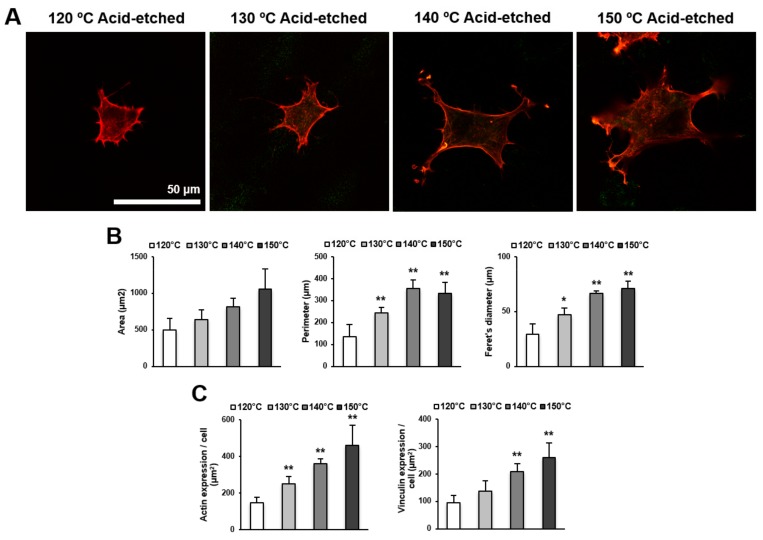
Representative confocal microscopy images of the spreading behavior of osteoblasts 24 h after seeding on acid-etched titanium discs at different temperatures. (**A**) The cells were stained with rhodamine phalloidin for actin filaments (red) and anti-vinculin antibody for vinculin (green). Scale bar = 50 μm. (**B**) Histograms for cytomorphometric parameters measured from the images. (**C**) The expression levels of actin and vinculin were semiquantified using the confocal microscopy images. Data are mean ± standard deviation (*n* = 5) for panels (B,C). * *p* < 0.05, ** *p* < 0.01, with a statistically significant difference when compared to the 120 °C acid-etched surface.

We next examined the spreading behavior of osteoblasts using higher magnification confocal microscopy images (Figure 4A,B). Cells appeared larger on titanium surfaces acid-etched at higher temperatures. In addition to the increased size of cells, the lamellipodia- and filopodia-like cytoskeletal projections were more advanced on these surfaces. These observations were confirmed by cytomorphometry results showing the increased area, perimeter, and Feret’s diameter of the cells. Image-based densitometry showed that the expression of cytoskeletal actin and vinculin—a focal adhesion protein—tended to increase with temperature (Figure 4C).

### 2.4. Osteoblast Differentiation

**Figure 5:**
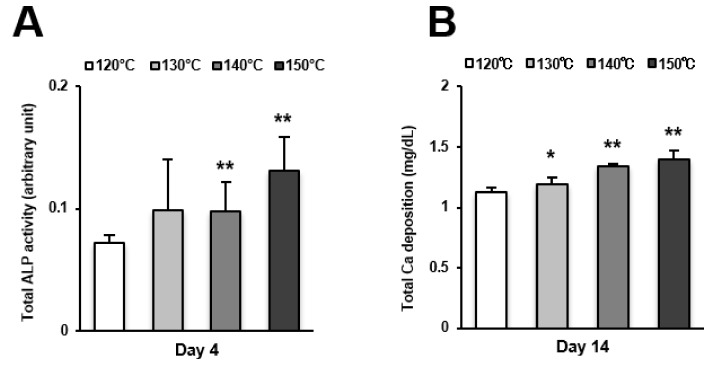
Osteoblast differentiation on titanium surfaces etched at different temperatures. (**A**) Alkaline phosphatase (ALP) activity at day 4, colorimetrically quantified and standardized relative to cell number. (**B**) Total calcium deposition at day 14, as measured using a colorimetry-based method. Each value represents the mean ± standard deviation of triplicate experiments (*n* = 3). * *p* < 0.05, ** *p* < 0.01, with a statistically significant difference when compared to the 120 °C acid-etched surface.

**Figure 6:**
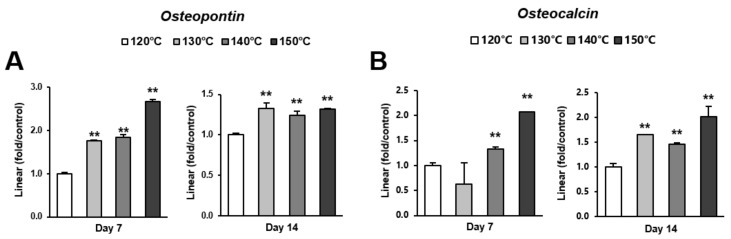
Gene expression levels of selected markers quantified via real-time qPCR. The osteogenic markers (**A**) osteopontin and (**B**) osteocalcin were analyzed. Total RNA was isolated at 7 and 14 days using osteoblastic cell cultures on titanium surfaces acid-etched at different temperatures. Relative expression levels (2^-ΔΔCt^ values) of the genes of interest were normalized to that of the housekeeping gene glyceraldehyde-3-phosphate dehydrogenase (*Gapdh*). Each value represents the mean ± standard deviation of triplicate experiments (*n* = 3). ** *p* < 0.01, with a statistically significant difference when compared to the 120 °C acid-etched surface.

Osteoblast differentiation was examined by alkaline phosphatase (ALP) activity, calcium deposition, and the expression of osteoblastic genes. ALP activity and calcium deposition increased with temperature (Figure 5A,B). The expression of osteopontin (*Opn*) increased on the surfaces acid-etched at higher temperatures on both day 7 (early stage of culture) and 14 (later stage of culture) (Figure 6A,B). This upregulated trend with increasing temperature was similar for osteocalcin (*Ocn*) expression, indicating that the three-scale roughness not only accelerated the differentiation of osteoblasts but also elevated the degree and probability of differentiation.

### 2.5. Biomechanical Strength of Bone-Implant Integration

**Figure 7:**
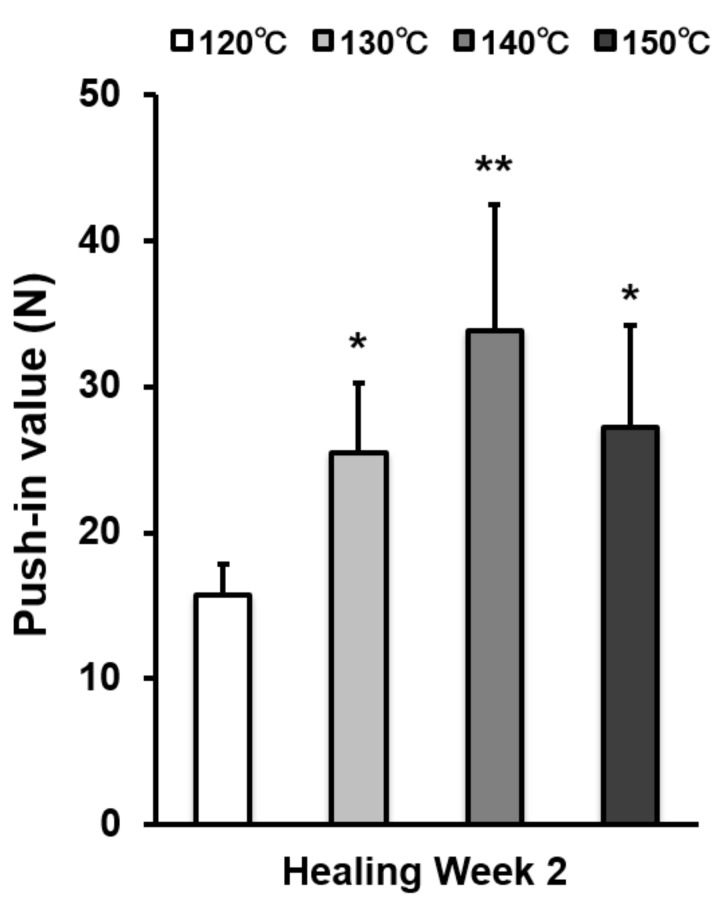
The strength of implant anchorage in bone, evaluated by the biomechanical push-in test in a rat femur model. Each bar represents mean ± standard deviation of titanium surfaces acid-etched at different temperatures (*n* = 6). * *p* < 0.05, ** *p* < 0.01, with a statistically significant difference when compared to the 120 °C acid-etched surface.

The strength of bone–titanium integration was measured by push-in value at week 2 (Figure 7). The push-in value increased with temperature during acid-etching and peaked at 140 °C. The push-in value of the 140 °C acid-etched surface was 2.3 times greater than that of the 120 °C-acid-etched surface.

### 2.6. Topographical Determinants for Osteoconductivity and Osseointegration

**Figure 8:**
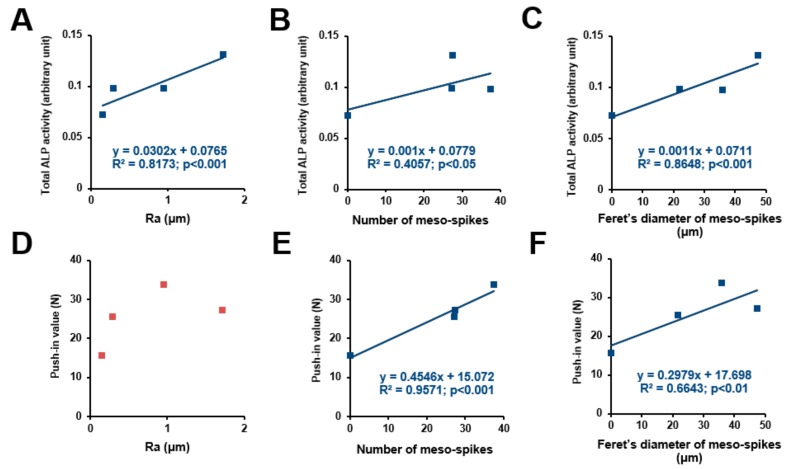
Correlation between differentiation ability or push-in value and each surface roughness parameter. Total ALP activity after incubation for 4 days plotted against the average roughness (Ra) of the acid-etched titanium surfaces, showing a significant linear correlation (**A**). Number of meso-spikes (**B**) and Feret’s diameter of meso-spikes (**C**) plotted against total ALP activity at day 4, showing a significant linear correlation between ALP activity and number of meso-spikes (B) and between ALP activity and Feret’s diameter of meso-spikes (C). Push-in value plotted against Ra, showing no significant correlation (**D**). Number of meso-spikes (**E**) and Feret’s diameter of meso-spikes (**F**) plotted against push-in value, showing a significant linear correlation between push-in value and number of meso-spikes (**E**) and between push-in value and Feret’s diameter of meso-spikes (F).

We sought potential topographical variables to determine the osteoconductivity and osseointegration abilities of our uniquely-created titanium surfaces. ALP activity (Figure 8A–C) and push-in values (Figure 8D–F) were plotted against the different surface variables’ Ra, number of meso-spikes, and Feret’s diameter of meso-spikes to extract their potential correlation (Figure 8). ALP activity increased linearly with all three surface variables—the higher the magnitude of the surface variables, the higher the ALP activity. The coefficient of determination (*R*^2^) was found between the ALP activity and the values for Feret’s diameter of the meso-spikes. The push-in value correlated significantly with the number of meso-spikes and their Feret’s diameters but not with Ra. Of note, the push-in values correlated with the number of meso-spikes with a remarkably high coefficient of determination.

## 3. Discussion

In the present study, we created the surface topography of three phases—meso-, micro-, and nano-scale—of a rough titanium surface using a simple and effective method. Across the surface of the acid-etched titanium, the micro-scale roughness was revealed in SEM images 1–5 μm wide. The meso-scale roughness was illustrated in three-dimensional scanning electron microscopy (3D-SEM) images 10–50 μm wide and 10–20 μm deep. As the temperature of the acid-etching rose, the height and width of the meso-scale structure as well as the number of structures increased. In addition, on the surface of the meso-scale structure, the nano-scale structure was observed with a width of approximately 100 nm and a rounded tip.

Results of our in vitro study showed a similar rate of osteoblast proliferation based on the different temperatures applied to acid-etched groups. In contrast, cell differentiation increased in the high temperature group. The rough surface induced osteoblastic phenotypes through increased ALP activity and upregulation of the expression levels of bone-related genes [35,36]. The results of the present study also showed that cell differentiation rose proportionally with the rise in temperature of acid-etching—in other words, with the increase in surface roughness. Moreover, the osteoblasts interacted with the titanium surface through integrin [37]. The promotion of differentiation at an early stage was indicated by confocal microscopy after 24 h of culture. These results agree with the behavior of osteoblasts that has been widely identified in previous studies [38,39,40,41]. In principle, the rates of osteoblast proliferation and differentiation are inversely correlated [38,41], i.e., when osteoblasts are robust in proliferative activity, their differentiation slows down and vice versa. This is partly because opposing growth factors regulate osteoblast proliferation and differentiation [42,43]. This biological discrepancy also applies to osteogenic cells at biomaterial and implant surfaces [19,44]. It has been reported that the proliferation of osteoblasts on roughened titanium surfaces is one-third to one-fifth of that observed on machined titanium surfaces [20,35,45,46]. It is notable that meso-, micro-, and nano-scale rough titanium (especially the 140 °C and 150 °C groups) did not reduce the rate of proliferation compared to the 120 °C acid-etched surface, although it did enable faster differentiation. Accelerating bone formation around implants is very important for clinical applications in both dental and orthopedic fields. In addition to the results of our in vitro study mentioned above, upregulated osteogenic gene expression was observed on the high temperature acid-etched surfaces, i.e., meso-, micro-, and nano-scale rough titanium. The late-stage markers such as *Opn* and *Ocn* were significantly upregulated, not only on day 7, but also on day 14. These gene expression results are also consistent with the mineralization assay results in vitro. The mineralization culture started on day 14 in our culture system. The present results indicate that differentiation and maturation of the osteoblasts was promoted. Previous studies report that nano-nodular titanium surfaces exhibit osteoblast proliferation rates similar to those observed in the present study, despite the substantial increase in overall surface roughness [12,47]. Furthermore, it has been reported that nano-scale structure also promotes osteoblast differentiation [25,26]. The mechanism underlying this observation requires further study; however, the rounded peaks of the nano-scale structure (compared to the sharp peaks typically seen on acid-etch-created rough titanium) might minimize the negative impact on osteoblast proliferation.

The strength of the integration was evaluated by an in vivo push-in test. The results showed that the strength was significantly increased as the temperature of the acid etching rose. This trend reached a peak at 140 °C and slightly decreased at 150 °C. The biological advantage of meso-, micro-, and nano-scale rough titanium surface, as consequently shown in the enhanced strength of bone–titanium integration, may not be explained solely by the increased surface area of titanium and increased bone–titanium interlocking. This corroborated the in vitro results that osteoblast proliferation and differentiation were upregulated on the meso-, micro-, and nano-scale rough titanium surface, indicating that bone–implant integration was not merely expedited but also enhanced. We assume that the combined effect of the increased bone–titanium mechanical interlocking and the enhanced function of osteoblasts is responsible for the enhanced strength of bone–titanium integration in which the nano-scale polymorphic structures regulated osteoblast function but not the mechanical interlocking. Moreover, in order to investigate the influence of each surface parameter on the differentiation or integration ability, we calculated a correlation. The result indicated that Ra showed positive correlation between the differentiation ability. It is well known that rougher surfaces induce osteoblast differentiation [20,44]. Interestingly, the number and size of the meso-scale structures, i.e., spike structure, had an effect on osseointegration ability. Therefore, it is assumed that the meso-scale structure not only improves the osseointegration ability as evaluated by the push-in test but also strengthens the mechanical interlocking of bone. Further research should be undertaken to reveal the physical and chemical properties involved in these processes.

In this study, we did not perform any morphological or biological analyses involving machined surfaces. The main focus of this study was the further enhancement of the rough titanium surface. This is because machined surface titanium implants are currently rarely used in clinics. The machined surface does not have any specific morphological feature apart from a pattern of parallel traces generated by the machining process [19,48]. It has been shown that titanium implants used in clinical practices can integrate into the bone much better when the implant surface shows some micro-roughness rather than being machined [49,50,51,52]. In addition, implant threads with micro-rough surfaces are also known to have a stronger mechanical interlocking force than those with the machined surface [52,53,54,55]. Evaluation of various previously reported morphological, physiochemical, and biological studies on machined and acid-etched surfaces [20,37,44,56,57,58,59,60] revealed that the three-scale rough surface generated in this study is clearly different from the machined surface.

The mechanism underlying the formation of the three-scale roughness needs to be studied further. The surface was made by a subtraction method via acid-etching. Different than the conventional micro-rough surface, the surface presented here has meso- and nano-structures. The procedure used in this study was simple, and no chemical other than sulfuric acid was used. Therefore, we postulate that a similar chemical reaction as in the conventional acid-etching occurred during the formation of the three-scale roughness. However, the mechanism underlying the typical micro-rough titanium surface made by simple acid-etching and most commonly used in dental implants remains unknown as well.

Here, we introduced an alternative titanium rough surface created by a simple method that demonstrated an improvement in osseointegration ability. This meso-, micro-, and nano-scale rough titanium is unique in terms of promoting osteoblast differentiation while maintaining proliferation at the same rate as the 120 °C acid-etched surface. Our results suggest that improvements in future implants are possible by optimizing meso-, micro-, and nano-scale surface topography. Furthermore, the technique would be applicable in clinical settings because the process is simple and does not require additional material. That being said, further studies to optimize the meso-, micro-, and nano-scale topography are necessary to increase their potential for routine use in medical and dental treatments. The surface presented in this study is novel and ground-breaking because of its three-scale structures that are simultaneously formed and its very simple, one-step manufacturing process. Therefore, this technology is expected to be utilized in dental and various types of orthopedic surgical implants, as well as titanium-based bone engineering scaffolds. Studies involving mid- and large-scale animals and human clinical trials are warranted.

## 4. Materials and Methods 

### 4.1. Titanium Samples and Surface Analysis

Titanium samples were prepared for cylindrical implants (1 mm in diameter, 2 mm in length) and disks (20 mm in diameter, 1.5 mm in thickness) of commercially pure grade 2 titanium (N: ≤ 0.03%, C: ≤ 0.08%, H: ≤ 0.013%, Fe: ≤ 0.20%, O: ≤ 0.15%, Ti: Balance) (Nishimura Kinzoku Co., Sabae, Fukui, Japan). Titanium surfaces were acid-etched with 67% H_2_SO_4_ for 75 s at 120, 130, 140, and 150 °C. The surface morphology of these surfaces was examined by scanning electron microscopy (SEM; XL30; Phillips, Eindhoven, Netherlands). Three-dimensional scanning electron microscopy (3D-SEM) images were built from SEM images using photogrammetry technique software (MeX; Alicona Imaging GmbH, Raaba/Graz, Austria). The average roughness (Ra) and the peak-to-valley height roughness (Rz) were calculated. The mean number of meso-scale spike structures per unit (/10^4^ µm^2^) and their diameters were examined using an image analyzer (ImageJ; NIH, Bethesda, MD, USA).

### 4.2. Osteoblastic Cell Culture

As established previously [61], bone marrow cells isolated from the femurs of 8-week-old male Sprague-Dawley rats were placed into alpha-modified Eagle’s medium supplemented with 15% fetal bovine serum, 50 mg/mL ascorbic acid, 10 mM Na-β-glycerophosphate, 10^-8^ M dexamethasone, and antibiotic-antimycotic solution. Cells were incubated in a humidified atmosphere of 95% air and 5% CO_2_ at 37 °C. At 80% confluency, the cells were detached using 0.25% trypsin-1 mM EDTA-4 Na and seeded onto the titanium disks at a density of 3 × 10^4^ cells/cm^2^. The culture medium was renewed every 3 days.

### 4.3. Cell Attachment Assay

Initial attachment of cells was evaluated by measuring the number of cells attached to titanium disks after 24 h of incubation using WST-1-based colorimetry (WST-1; Roche Applied Science, Mannheim, Germany). The culture well was incubated with 100 µL WST-1 reagent at 37 °C for 4 h. The amount of formazan produced was measured using an enzyme-linked immunosorbent assay (ELISA) plate reader (Synergy HT; Bio Tek Instruments, Inc., Winooski, VT, USA) at 450 nm.

### 4.4. Cell Morphology and Morphometry

Confocal laser scanning microscopy (TCS SP5; Leica, Wetzlar, Germany) was used to examine cell morphology and cytoskeletal arrangement of osteoblasts seeded onto the titanium surfaces. After 24 h of culture, cells were fixed in 10% formalin solution and stained using the fluorescent dye rhodamine phalloidin (actin filament, red color; R415; Molecular Probes, Eugene, OR, USA). The area, perimeter, and Ferret’s diameter of cells were quantified using an image analyzer (ImageJ).

### 4.5. Vinculin and Actin Expression Analysis

The expression and localization of the focal adhesion protein vinculin were analyzed by microscopy image-based observation and densitometry at 24 h of culture. During preparation for the confocal microscopy analysis, the cultures were also immunochemically stained with mouse anti-vinculin monoclonal antibody (green color; ab11149; Abcam, Cambridge, MA, USA), followed by FITC-conjugated anti-mouse secondary antibody (Abcam). The level of vinculin expression was quantified as a pixel-based density using an image analyzer (ImageJ). Densitometry was also applied to quantify the expression of actin filaments in the same manner using the images stained with rhodamine phalloidin.

### 4.6. Alkaline Phosphatase (ALP) Activity

The ALP activity of osteoblasts was examined at day 4 using a colorimetry-based assay. The culture was rinsed with double-distilled water (ddH_2_O) and treated with 250 µL p-nitrophenylphosphate (LabAssay ALP, Wako Pure Chemicals, Richmond, VA, USA) and further incubated at 37 °C for 15 min. The ALP activity was evaluated as the amount of nitrophenol released through the enzymatic reaction and measured at a wavelength of 405 nm using an ELISA plate reader (Synergy HT). 

### 4.7. Mineralization Assay

The mineralization capability of cultured osteoblasts was examined by colorimetry-based quantification of calcium deposition at day 14. The cultures were washed with PBS and incubated overnight in 1 mL of 0.5 mM HCl solution with gentle shaking. The solution was mixed with *o*-cresolphthalein complexone in an alkaline medium (calcium binding and buffer reagent; Sigma-Aldrich, St. Louis, MO, USA) to produce a red calcium cresolphthalein complexone complex. Color intensity was measured by an ELISA plate reader (Synergy HT) at 575 nm absorbance.

### 4.8. Real-Time Quantitativepcr (Real-Time qPCR)

Total RNA was isolated at days 7 and 14 using an RNeasy Plus Mini Kit (Qiagen, Hilden, Germany) and complementary DNA (cDNA) was made as described previously [62]. Real-time qPCR was performed in triplicate for each sample with LC480 SYBR Green I Master (Roche Diagnostics, Indianapolis, IN, USA) using universal cycling conditions on a LightCycler 480 (Roche) [63]. A total of 55 cycles were executed and the second derivative Cq value determination method was used to compare fold-differences. For PCR amplification, the target-specific PCR primers for osteopontin (*Opn*), osteocalcin (*Ocn*), and glyceraldehyde-3-phosphate dehydrogenase (*Gapdh*), a housekeeping gene, were used (Table 1). Analysis of relative gene expression was performed with the 2^-ΔΔCt^ method [19]. The expression levels of various genes were expressed as fold differences in gene expression relative to that of the 120 °C acid-etched surface.

### 4.9. Implant Surgery

Eight-week-old male Sprague-Dawley rats were anesthetized with 1%–2% isoflurane inhalation. After their legs were shaved and scrubbed with 10% povidone-iodine solution, the distal aspects of the femurs were carefully exposed via skin incision and muscle dissection. The flat surfaces of the distal femurs were selected for implant placement. The implant site was prepared 9 mm from the distal edge of the femur by drilling with a 0.8 mm round burr and enlarged using reamers (#ISO 090 and 100). Profuse irrigation with sterile isotonic saline solution was used for cooling and cleaning. One titanium cylindrical implant, acid-etched at different temperatures (1 mm in diameter and 2 mm in length), was placed into the prepared hole. Surgical sites were then closed in layers. Muscle and skin were sutured separately with resorbable suture thread. The University of California at Los Angeles (UCLA) Chancellor’s Animal Research Committee approved this protocol (ARC #2005-175-41E, approved on 30 January 2018) and all experimentation was performed in accordance with the United States Department of Agriculture (USDA) guidelines on animal research.

### 4.10. Biomechanical Implant Push-In Test

The biomechanical implant push-in test was used to assess the biomechanical strength of bone-implant integration, as described previously [48,64]. At week 2 of healing, femurs containing a cylindrical implant were harvested and embedded into auto-polymerizing resin at the top surface of the implant level. A micro-CT machine (μCT 40; SancroMedica, Bassersdorf, Switzerland) was used to confirm that the implants were free from cortical bone support from the lateral and bottom sides of the implant. The testing machine (Instron 5544 electromechanical testing system; Instron, Norwood, MA, USA) equipped with a 2000 N load cell and a pushing rod (diameter 1⁄4 0.8 mm) was used to load the implant vertically downward at a crosshead speed of 1 mm/min. The push-in value was determined by measuring the peak of the load–displacement curve.

### 4.11. Statistical Analysis

We used three samples for the cell culture studies, except for the surface analysis which was evaluated in five samples; five were used for cell morphometry and the number of implants for the implant push-in test was six. Statistical analysis was carried out using SPSS Version 22.0 (IBM Corp., Armonk, NY, USA). One-way ANOVA was used to examine the effects of titanium surfaces acid-etched at different temperatures; *p* < 0.05 was considered statistically significant. Correlations between total ALP activity and titanium surface topography, and push-in value and surface topography were examined; regression formulas were determined by least-squares mean approximation.

## 5. Conclusions

We created a novel titanium surface with meso-, micro-, and nano-scale roughness by employing high temperature acid-etching. This three-scale rough titanium significantly promoted osteoblast differentiation without compromising the attachment and spreading of osteoblasts. Osteoconductivity and osseointegration ability were highly correlated with the size and density of meso-scale spikes. The overall strength of osseointegration was the greatest when the acid-etching was performed at 140 °C, and the strength was 2.3 times greater than that of a titanium surface with micro-scale roughness alone.

## Figures and Tables

**Table 1:** Primer sequences for real-time qPCR. *Opn*: osteopontin, *Ocn*: osteocalcin.

*Gapdh*	Forward	AACCCATCACCATCTTCCAGG
	Reverse	GCCTTCTCCATGGTGGTGAA
*Opn*	Forward	GACAGCAACGGGAAGACC
	Reverse	CAGGCTGGCTTTGGAACT
*Ocn*	Forward	GAGGGCAGTAAGGTGGTGAA
	Reverse	CGTCCTGGAAGCCAATGTG

## Abbreviations

ALP	Alkaline phosphatase
ANOVA	One-way analysis of variance
cDNA	Complementary DNA
ELISA	Enzyme-linked immunosorbent assay
Gapdh	Glyceraldehyde-3-phosphate dehydrogenase
Ocn	Osteocalcin
Opn	Osteopontin
PCR	Polymerase chain reaction
SEM	Scanning electron microscopy
WST	Water soluble tetrazolium salts
